# 新辅助免疫治疗联合化疗与手术治疗局部晚期可切除非小细胞肺癌的短期疗效比较

**DOI:** 10.3779/j.issn.1009-3419.2024.102.26

**Published:** 2024-06-20

**Authors:** Haitian LI, Qing LIU, Bin LI, Yuzhen CHEN, Junping LIN, Yuqi MENG, Haiming FENG, Zhizhong ZHENG, Yiming HUI

**Affiliations:** 730030 兰州，兰州大学第二医院（第二临床医学院）胸外科; Department of Thoracic Surgery, The Second Hospital & Clinical Medical School, Lanzhou University, Lanzhou 730030, China

**Keywords:** 肺肿瘤, 新辅助免疫化疗, PD-1抑制剂, 客观缓解率, 并发症, Lung noeplasms, Neoadjuvant immunochemotherapy, PD-1 inhibitors, Objective response rate, Complication

## Abstract

**背景与目的** 肺癌是中国发生率和死亡率均排名第一的癌症，非小细胞肺癌（non-small cell lung cancer, NSCLC）占所有肺恶性肿瘤的80%-85%。目前，手术治疗仍是肺癌主要的治疗方式。近年来，免疫检查点抑制剂在NSCLC中的疗效已成为共识，新辅助免疫联合化疗（neoadjuvant immunochemotherapy, nICT）在早中期NSCLC中显示出来良好的疗效和安全性。然而，nICT治疗局部晚期NSCLC的相关研究较少。本研究旨在评估程序性死亡受体1（programmed cell death 1, PD-1）抑制剂联合含铂两药新辅助治疗治疗局部晚期可切除NSCLC的有效性和安全性。**方法** 纳入2021年1月至2024年4月于兰州大学第二医院胸外科就诊的85例确诊可切除IIIA、IIIB期患者，分为nICT组（n=32）和单纯手术组（n=53），比较两组患者的临床基线资料、围手术期相关指标及术后并发症，并评估nICT组的影像学缓解率、病理学缓解率、相关不良反应发生率、生活质量。**结果** 两组患者临床基线资料差异均无统计学意义（P>0.05）。nICT组选择开胸方式比单纯手术组发生率高（P=0.002），两组手术时间、术中出血量、清扫淋巴结个数、带管时间、术后住院时间及R0切除率的差异无统计学意义（P>0.05）。nICT组与单纯手术组术后总并发症发生率分别为31.25%和22.64%，差异无统计学意义（P=0.380）。nICT组中客观缓解率（objective response rate, ORR）为84.38%，完全缓解（complete response, CR）5例（15.63%），部分缓解（partial response, PR）22例（68.75%），病理性完全缓解（pathological complete response, pCR）15例（46.88%），主要病理缓解（major pathological reaponse, MPR）11例（34.38%）。nICT治疗期间，3级治疗相关不良反应共12例（37.50%），无不良反应或免疫相关不良反应导致患者死亡。而且，nICT治疗后患者相关症状有所改善。**结论** nICT治疗局部晚期可切除NSCLC显示出良好的疗效，治疗相关不良事件可控，是局部晚期可切除NSCLC安全、可行的新辅助治疗模式。

## Abstract (en)

**Background and objective** Lung cancer is the cancer with the highest incidence and mortality rates in China, and non-small cell lung cancer (NSCLC) accounts for 80%-85% of all malignant lung tumors. Currently, surgical treatment remains the primary treatment modality for lung cancer. In recent years, the effectiveness of immune checkpoint inhibitors for NSCLC has become a consensus, and neoadjuvant immunochemotherapy (nICT) has shown promising efficacy and safety in early to intermediate stage NSCLC. However, there are fewer studies related to nICT for locally advanced NSCLC. This study aims to evaluate the efficacy and safety of nICT therapy in locally advanced resectable NSCLC. **Methods** 85 confirmed resectable stage IIIA and IIIB patients treated in the Department of Thoracic Surgery, Second Hospital of Lanzhou University, from January 2021 to April 2024, were divided into the nICT group (n=32) and the surgery alone group (n=53). Clinical baseline data, perioperative indicators, postoperative complications, imaging response rate, pathological response rate, incidence of adverse events, and quality of life were compared between the two groups. **Results** There were no statistically significant differences in clinical baseline data between the two groups (P>0.05). Incidence of choosing thoracotomy was higher in the nICT group than in the surgery alone group (P=0.002). There were no significant differences in surgical time, intraoperative blood loss, number of dissected lymph nodes, duration of chest tube placement, postoperative hospital stay, and R0 resection rate between the two groups (P>0.05). The overall incidence of postoperative complications was 31.25% in the nICT group and 22.64% in the surgery alone group, with no statistically significant difference (P=0.380). In the nICT group, the objective response rate (ORR) was 84.38%, with 5 cases of complete response (CR)(15.63%), 22 cases of partial response (PR)(68.75%), 15 cases of pathological response rate (pCR)(46.88%), and 11 cases of major pathological reaponse (MPR) (34.38%). During nICT treatment, 12 cases (37.50%) experienced grade 3 treatment-related adverse events, no death induced by adverse events or immune related adverse events. Moreover, the symptoms of the patients were improved after nICT treatment. **Conclusion** Neoadjuvant immunochemotherapy shows promising efficacy in locally advanced resectable NSCLC, with manageable treatment-related adverse events. It is a safe and feasible neoadjuvant treatment modality for locally advanced resectable NSCLC.

在中国，肺癌是发生率和死亡率均居于第一的癌症^[1]^。在肺癌的组织类型中，非小细胞肺癌（non-small cell lung cancer, NSCLC）占80%-85%。手术治疗是可切除NSCLC的主要治疗方案，在接受手术的患者中，30%-70%的患者会发展为转移性疾病^[2]^，这也导致了肺癌患者的5年生存率较低。在过去的二十年内，新辅助含铂两药化疗方案逐渐进入临床，然而，荟萃分析^[3,4]^结果显示NSCLC患者新辅助含铂两药5年绝对生存率仅仅提高了4%-5%，亟需新的治疗方式。近年来，程序性细胞死亡受体1（programmed cell death 1, PD-1）单抗联合化疗在NSCLC治疗中取得了显著的疗效，CheckMate 816研究^[5]^是首个在IB-IIIA期可切除NSCLC中取得阳性结果的免疫新辅助III期临床研究，随着免疫治疗联合方案研究结果相继公布，新辅助化疗联合免疫治疗（neoadjuvant immunochemotherapy, nICT）具有更好的有效性及安全性。本研究中，我们回顾性分析兰州大学第二医院胸外科收治的可切除IIIA-IIIB期NSCLC患者85例，分析新辅助免疫联合含铂两药和单纯手术对于NSCLC的有效性及安全性，为局部晚期NSCLC术前治疗方案的选择提供参考。

## 1 资料与方法

### 1.1 临床资料

回顾性分析2021年1月至2024年4月就诊于兰州大学第二医院胸外科确诊可切除IIIA、IIIB期单纯手术以及接受新辅助免疫联合化疗的NSCLC患者，依据美国癌症联合会（American Joint Committee on Cancer, AJCC）肿瘤原发灶-淋巴结-转移（tumor-node-metastasis, TNM）分期（第8版）进行肿瘤分期^[6]^。

纳入标准：（1）确诊NSCLC，根据胸部增强计算机断层扫描（computed tomography, CT）影像学进行评估，对于可疑转移病灶，进行多学科讨论及正电子发射计算机断层显像（positron emission computed tomography/CT, PET/CT）检查及进行评估分期，分期为IIIA、IIIB期，年龄18-75岁，性别不限；（2）根据实体瘤疗效评价标准1.1版（Response Evaluation Criteria in Solid Tumour 1.1, RECIST 1.1）^[7]^，至少有一处影像学可测量病灶；（3）美国东部肿瘤协作组（Eastern Cooperative Oncology Group, ECOG）卡氏体能状态评分0-1分；（4）肝肾功能基本正常、心肺功能良好，确认符合出于根治性治疗为目的进行手术切除的要求。

排除标准：（1）首次给药前5年内诊断为其他恶性肿瘤且未治愈的患者；（2）具有间变性淋巴瘤激酶（anaplastic lymphoma kinase, ALK）、表皮生长因子受体（epidermal growth factor receptor, EGFR）突变的患者；（3）具有远处转移或存在手术禁忌证的患者；（4）既往接受过抗PD-1、抗程序性死亡配体1（programmed cell death ligand 1, PD-L1）治疗的患者；（5）患有自身免疫性疾病，或长期应用免疫抑制类药物的患者；（6）妊娠或哺乳期妇女。本研究已通过医院医学伦理委员会审批（批件文号：2023A-596）。

### 1.2 治疗方案

免疫药物均为PD-1抑制剂，包括替雷利珠单抗（200 mg, ivgtt, d1）、信迪利单抗（200 mg, ivgtt, d1）。化疗方案：腺癌选择培美曲塞（500 mg/m^2^, ivgtt, d1）+顺铂（75 mg/m^2 ^, ivgtt, d1）/卡铂[曲线下面积（area under the curve, AUC）=5，ivgtt，d1]；鳞癌选择紫杉醇（175 mg/m^2^, ivgtt, d1）+顺铂（75 mg/m^2^, ivgtt, d1）/卡铂（AUC=5, ivgtt, d1）。nICT治疗21 d为1个周期，用药2-3个周期后间隔4-6周评估nICT疗效并进行手术。

### 1.3 观察指标

#### 1.3.1 手术相关数据

记录手术患者的手术时间、术中出血量、术中淋巴结清扫个数、R0切除率、带管时间、术后住院时间和术后并发症等。术后并发症根据2017年发布的《胸外科疾病标准化诊疗术语》及国际通用外科并发症Clavien-Dindo分级^[8]^的建议进行诊断和定义。

#### 1.3.2 nICT疗效评价

nICT治疗前及手术前进行胸部CT检查，根据RECIST 1.1对影像学疗效进行评估，分为完全缓解（complete response, CR）：所有靶病灶完全消失；部分缓解（partial response, PR）：所有可测量靶病灶的直径总和缩小幅度≥30%；疾病稳定（stable disease, SD）：治疗后病灶与治疗前比较无明显增大或缩小；疾病进展（progressive disease, PD）：治疗后病灶增大20%，病情未达到CR、PR或SD标准，并记录客观缓解率（objective response rate, ORR），即CR+PR所占比例。nICT组手术标本评估依据国际肺癌研究协会（International Association for the Study of Lung Cancer, IASLC）多学科建议^[9]^分为病理完全缓解（pathological CR, pCR）和主要病理缓解（major pathological response, MPR），MPR指术后标本病理检测残留肿瘤细胞≤10%。

#### 1.3.3 nICT治疗相关不良事件（adverse events, AEs）评估

自nICT治疗开始至结束后1个月内，记录所有AEs，无论与治疗药物是否存在因果关系，均视为AEs。依据常见不良事件评价标准（Common Terminology Criteria for Adverse Events, CTCAE）5.0版^[10]^评估AEs的发生率和严重程度，特别是免疫相关AEs。

#### 1.3.4 nICT生活质量评估

用于评估肺癌患者生活质量的标准问卷由欧洲癌症研究与治疗组织（European Organisation for Research and Treatment of Cancer, EORTC）开发的癌症患者生命质量测量表体系中的核心量表（quality of life questionnaire-core 30, QLQ-C30）^[11]^以及肺癌特异性模板（quality of life lung cancer questionnaire-core 13, QLQ-LC13）^[12]^组成。研究^[13]^表明，EORTC QLQ-C30和QLQ-LC13在肺癌生活质量调查评估中具有良好的信度和效度，在临床中广泛运用。EORTC QLQ-C30和QLQ-LC13的原始分数经线性公式转换为0-100分^[14]^。对于功能量表，分数越高表示功能水平越高，对于症状量表和症状单项，分数越高表示症状越多或越差。该生活质量评估与nICT知情同意书一同完成，评估通过纸质版调查问卷进行，共发放问卷32份，收回29份完整调查问卷。

### 1.4 统计学方法

数据采用SPSS 27.0软件完成统计学分析。分类变量用频数和百分比描述，组间比较采用χ^2^检验或Fisher’s精确概率法；正态分布的连续变量用均数±标准差（Mean±SD）表示，组间比较采用t检验。非正态分布资料采用中位数（P25, P75）进行统计描述，组间比较采用Wilcoxon秩和检验分析。P<0.05为差异有统计学意义。

## 2 结果

### 2.1 一般资料

共纳入患者107例，nICT组54例，单纯手术组53例，其中nICT组32例患者完成手术治疗，其余22例患者完成了2-3个周期新辅助治疗（8例待术，6例随访丢失，4例因影像学评估CR后拒绝手术，4例因家庭经济原因拒绝手术），最终纳入患者85例。nICT组中接受2个周期治疗7例，接受3个周期25例；接受替雷利珠单抗联合含铂两药组18例，接受信迪利单抗联合含铂两药组14例。两组患者临床资料间无统计学差异（P>0.05），见表1。

**表1:** 两组患者一般临床资料比较

Characteristics		nICT group (n=32)	Surgery alone group (n=53)	t/χ^2^	P
Age (yr)		59.44±6.72	57.32±9.06	1.144	0.256
Gender	Female	4 (12.50%)	16 (30.19%)	3.470	0.063
	Male	28 (87.50%)	37 (69.81%)		
Histology	Adenocarcinoma	9 (28.13%)	25 (47.17%)	3.015	0.082
	Squamous carcinoma	23 (71.88%)	28 (52.83%)		
Smoking history	No	14 (43.75%)	33 (62.26%)	2.767	0.096
	Yes	18 (56.25%)	20 (37.74%)		
Alcohol history	No	24 (75.00%)	45 (84.91%)	1.281	0.258
	Yes	8 (25.00%)	8 (15.09%)		
Hypertension	No	27 (84.38%)	40 (75.47%)	0.948	0.330
	Yes	5 (15.63%)	13 (24.53%)		
Diabetes mellitus	No	30 (93.75%)	49 (92.45%)	0.051	0.821
	Yes	2 (6.25%)	4 (7.55%)		
ASA classification	I	1 (3.13%)	0 (0.00%)	2.322	0.313
	II	27 (84.38%)	49 (92.45%)		
	III	4 (12.50%)	4 (7.55%)		
Tumor location	Right lung upper lobe	7 (21.88%)	14 (26.42%)	8.353	0.079
	Right lung lower lobe	12 (37.50%)	15 (28.30%)		
	Right lung middle lobe	0 (0.00%)	5 (9.43%)		
	Left lung upper lobe	9 (28.13%)	6 (11.32%)		
	Left lung lower lobe	4 (12.50%)	13 (24.53%)		
T stage	T1c	3 (9.38%)	17 (32.08%)	8.137	0.087
	T2a	6 (18.75%)	13 (24.53%)		
	T2b	5 (15.63%)	5 (9.43%)		
	T3	11 (34.38%)	9 (16.98%)		
	T4	7 (21.88%)	9 (16.98%)		
N stage	N1	0 (0.00%)	2 (3.77%)	1.237	0.266
	N2	32 (100.00%)	51 (96.23%)		
Clinical stage	IIIA	17 (53.13%)	38 (71.70%)	3.014	0.083
	IIIB	15 (46.88%	15 (28.30%)		

nICT: neoadjuvant immunochemotherapy; ASA classification: American Society of Anesthesiologists Physical Status classification.

### 2.2 围手术期指标

与单纯手术组相比，nICT组开胸手术12例（37.50%），其中中转开胸2例（16.67%）；单纯手术组开胸手术5例（9.43%），其中中转开胸2例（40.00%）。nICT组选择开胸方式比单纯手术组发生率高（P=0.002）。两组手术时间、术中出血量、清扫淋巴结个数、带管时间、术后住院天数及R0切除率的差异均无统计学意义（P>0.05），其中，nICT组R0切除率达100.00%，见表2。

**表2:** 两组可切除手术患者围手术期相关指标比较

Characteristics		nICT group (n=32)	Surgery alone group (n=53)	Z/χ^2^	P
Surgery time (min) [Median (P25, P75)]		162.50 (130.00, 180.00)	180.00 (120.00, 180.00)	0.127	0.899
Intraoperative blood loss (mL) [Median (P25, P75)]		50.00 (45.00, 100.00)	50.00 (30.00, 100.00)	0.444	0.657
Number of lymph nodes cleared [Median (P25, P75)]		17.00 (12.00, 26.00)	21.00 (14.00, 27.00)	1.774	0.776
Duration of intubation (d) [Median (P25, P75)]		5.00 (3.00, 6.00)	5.00 (3.00, 7.00)	1.074	0.283
Postoperative hospital stay (d) [Median (P25, P75)]		6.00 (5.00, 7.25)	6.00 (4.00, 8.00)	0.456	0.649
R0 resection	Yes	32 (100.00%)	50 (94.34%)	1.878	0.171
	No	0 (0.00%)	3 (5.66%)		
Surgical approach or technique	VATS	20 (62.50%)	48 (90.57%)	9.823	0.002
	Open surgery	12 (37.50%)	5 (9.43%)		

R0 resection: complete resection with clear margins; VATS: video-assisted thoracic surgery.

nICT组与单纯手术组术后并发症发生率无统计学差异（P>0.05）。本研究中，术后总并发症发生率分别为31.25%和22.64%，其中，肺炎发生率最高，见表3。

**表3:** 两组可切除患者术后并发症资料比较

Characteristics	nICT group (n=32)	Surgery alone group (n=53)	χ^2^	P
Overall complications	10 (31.25%)	12 (22.64%)	0.771	0.380
Pulmonary infection	8 (25.00%)	9 (16.98%)	0.802	0.371
Arrhythmia	7 (21.88%)	8 (15.09%)	0.631	0.427
Pneumothorax	6 (18.75%)	5 (9.43%)	1.537	0.215
Pleural effusion	5 (15.63%)	6 (11.32%)	0.328	0.567
Persistent air leak	2 (6.25%)	4 (7.55%)	0.051	0.821
Hemoptysis	3 (9.38%)	5 (9.43%)	0.000	0.993
Hoarseness	1 (3.13%)	2 (3.77%)	0.025	0.875
Respiratory failure	2 (6.25%)	1 (1.89%)	1.116	0.291
Heart failure	1 (3.13%)	0 (0.00%)	1.676	0.195

### 2.3 nICT组患者的疗效评估

nICT组患者手术前均根据RECIST 1.1评估，其中ORR率为84.38%，CR 5例（15.63%），PR 22例（68.75%），SD 4例（12.50%），PD 1例（3.13%）。手术后病理学疗效：pCR 15例（46.88%），MPR 11例（34.38%），总病理缓解率达81.25%，其中ypT0为15例（46.88%），ypN0为23例（71.88%）。鳞癌pCR为65.22%，MPR为21.74%；腺癌pCR为0.00%，MPR为66.67%。nICT前后pCR、MPR患者治疗前后胸部CT、气管镜及病理对比见图1、图2。

**图1:**
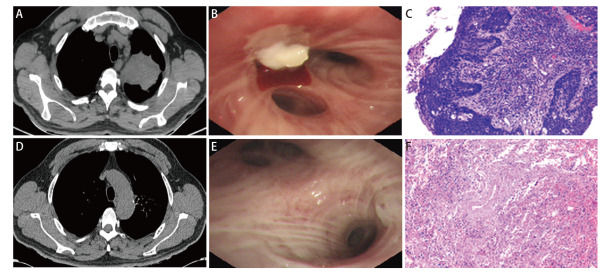
pCR患者nICT治疗前后胸部CT、气管镜、病理对比。nICT治疗前：A：胸部增强CT示左肺上叶占位；B：气管镜示左肺上叶固有支占位；C：气管镜活检示鳞状细胞癌。nICT治疗后；D：胸部CT示未见明显肿瘤存在；E：气管镜左肺上叶支气管急性炎症改变；F：术后病理示局灶纤维化，肺泡上皮未见异型增生。

**图2:**
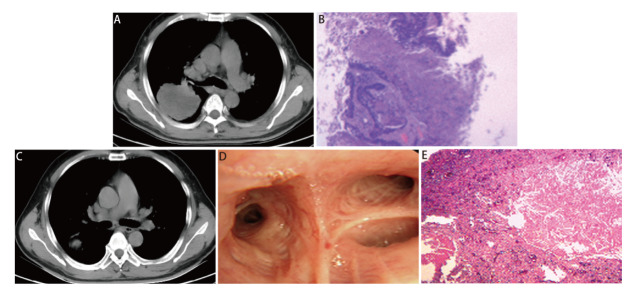
MPR患者nICT治疗前后胸部CT、病理对比。nICT治疗前：A：胸部增强CT示右肺上叶占位；B：肺穿刺活检示鳞状细胞癌。nICT治疗后：C：胸部CT示较前肿瘤退缩明显；D：气管镜右肺上叶正常；E：术后病理示瘤床区坏死显著，见少量活肿瘤细胞，支气管切缘未见癌组织残留。

### 2.4 nICT治疗相关AEs

本研究AEs多为1、2级，且多与化疗药物相关，根据CTCAE 5.0版，32例（100.00%）患者均发生了不同程度的药物AEs，乏力及脱发发生率最高，为68.75%，3级AEs共12例（37.50%），无AEs或免疫相关AEs导致患者死亡。见表4。

**表4:** nICT治疗有关AEs（n=29）

AEs	Grade 1-2	Grade 3
Fatigue	22 (68.75%)	0 (0.00%)
Alopecia	22 (68.75%)	0 (0.00%)
Nausea	21 (65.63%)	0 (0.00%)
Anorexia	14 (43.75%)	0 (0.00%)
Rash	14 (43.75%)	1 (3.13%)
Leukopenia	10 (31.25%)	4 (12.50%)
Anemia	11 (34.38%)	1 (3.13%)
Elevated liver enzymes	11 (34.38%)	0 (0.00%)
Thrombocytopenia	8 (25.00%)	1 (3.13%)
Diarrhea	8 (25.00%)	0 (0.00%)
Constipation	7 (21.88%)	0 (0.00%)
Vomiting	7 (21.88%)	0 (0.00%)
Neutropenia	5 (15.63%)	1 (3.13%)
Thyroid dysfunction	4 (12.50%)	2 (6.25%)
Transfusion reaction	5 (15.63%)	0 (0.00%)
Dizziness	4 (12.50%)	0 (0.00%)
Renal dysfunction	3 (9.38%)	0 (0.00%)
Cushing’s syndrome	3 (9.38%)	0 (0.00%)
Immune-related pneumonia	0 (0.00%)	2 (6.25%)

AEs: adverse events.

### 2.5 nICT治疗患者生活质量评估

 29例患者完成了nICT前后生活质量评估，结果显示，在QLQ-C30的功能量表中，躯体功能（P=0.006）、情绪功能（P=0.032）治疗前后有统计学差异，其余功能得分治疗前后均无统计学差异（P>0.05）。在QLQ-C30、QLC-LC13的症状领域量表中，患者接受nICT治疗后部分症状改善明显，但伴有治疗相关AEs的出现。患者疲乏、恶心与呕吐、失眠、经济困难、口腔溃疡、周围神经痛、脱发、手臂及肩膀痛等评分在nICT治疗后明显增加（P<0.05），而气促、咳嗽、咯血等评分在nICT治疗后明显改善（P<0.05），其余症状评分改变在nICT治疗前后不具有统计学差异（P>0.05）。见表5、表6。

**表5:** nICT治疗前后患者QLQ-C30量表评分比较（n=29）[Median (P25, P75)]

Characteristics	Before nICT treatment	After nICT treatment	Z	P
Physical function	86.67 (86.67, 93.33)	86.67 (80.00, 86.67)	2.743	0.006
Role function	83.33 (66.67, 100.00)	83.33 (66.67, 83.33)	1.077	0.281
Emotional function	91.67 (83.33, 100.00)	91.67 (83.33, 91.67)	2.150	0.032
Cognitive function	100.00 (83.33, 100.00)	100.00 (83.33, 100.00)	0.498	0.618
Social function	83.33 (66.67, 100.00)	83.33 (66.67, 100.00)	0.720	0.471
Global health status	66.67 (58.33, 75.00)	66.67 (58.33, 66.67)	1.122	0.262
Fatigue	11.11 (0.00, 22.22)	22.22 (11.11, 22.22)	2.809	0.005
Nausea and vomiting	0.00 (0.00, 0.00)	16.67 (0.00, 16.67)	3.974	<0.001
Pain	0.00 (0.00, 16.67)	0.00 (0.00, 16.67)	1.390	0.165
Dyspnea	33.33 (33.33, 66.67)	33.33 (0.00, 33.33)	3.386	0.001
Sleep disturbance	0.00 (0.00, 33.33)	33.33 (0.00, 33.33)	2.177	0.029
Appetite loss	0.00 (0.00, 33.33)	33.33 (0.00, 33.33)	1.941	0.052
Constipation	0.00 (0.00, 0.00)	0.00 (0.00, 0.00)	1.897	0.058
Diarrhea	0.00 (0.00, 0.00)	0.00 (0.00, 0.00)	0.333	0.739
Financial difficulties	33.33 (0.00, 33.33)	33.33 (33.33, 66.67)	3.506	<0.001

QLQ-C30: quality of life questionnaire-core 30.

**表6:** nICT前后患者QLQ-LC13量表评分的比较（n=29）[Median (P25, P75)]

Characteristics	Before nICT	After nICT	Z	P
Dyspnea	33.33 (11.11, 33.33)	22.22 (11.11, 33.33)	3.274	0.001
Cough	33.33 (33.33, 66.67)	33.33 (33.33, 33.33)	2.409	0.016
Haemoptysis	0.00 (0.00, 33.33)	0.00 (0.00, 0.00)	3.022	0.003
Sore mouth	0.00 (0.00, 0.00)	0.00 (0.00, 0.00)	2.333	0.020
Dysphagia	0.00 (0.00, 0.00)	0.00 (0.00, 0.00)	1.414	0.157
Peripheral neuropathy	0.00 (0.00, 0.00)	33.33 (0.00, 33.33)	3.557	<0.001
Alopecia	0.00 (0.00, 0.00)	66.67 (0.00, 66.67)	4.165	<0.001
Pain in chest	0.00 (0.00, 33.33)	33.33 (0.00, 33.33)	1.400	0.161
Pain in arm or shoulder	0.00 (0.00, 0.00)	0.00 (0.00, 33.33)	2.807	0.005
Pain in other part	0.00 (0.00, 0.00)	0.00 (0.00, 0.00)	1.732	0.083

QLQ-LC13: quality of life lung cancer questionnaire-core 13.

## 3 讨论

新辅助治疗的目的在于延长肿瘤患者生存期、提高R0切除率、减少肿瘤复发。近年来，nICT治疗的发展改变了NSCLC的治疗方式，nICT治疗展现出良好的临床应用前景，新近研究数据的公布为nICT方案提供了大量的支持^[15,16]^。目前，PD-1抑制剂联合化疗在早期NSCLC术前新辅助中取得了显著的疗效，然而对于局部晚期IIIA、IIIB期可切除NSCLC治疗上选择nICT治疗还是直接手术治疗存在争议，nICT治疗后取消手术、手术延迟、手术难度增加等是外科医生面对的问题^[17]^，而直接手术后肿瘤的复发转移、生存时间同样是我们需要面临的问题。

我们回顾性分析了兰州大学第二医院胸外科nICT治疗局部晚期可切除NSCLC与单纯手术患者的疗效及手术指标，研究结果表明，nICT治疗可显著改善肿瘤的影像学及病理学消退，ORR为84.38%，pCR为46.88%，MPR为34.38%，总病理缓解率达81.25%。相比于III期CheckMate 816研究^[5]^及KEYNOTE-671研究^[18]^，本研究取得了较高的影像学及病理学缓解率。有研究^[19]^表明，病理缓解与生存存在一定的关系，病理残余存活肿瘤越低，患者无事件生存期（event-free survivl, EFS）发生率越低且生存期越长。本研究显著的影像学缓解、病理缓解率为nICT治疗局部晚期可切除NSCLC建立了一定的基础。

众所周知，早期诊断和及时手术可显著提高肺癌的治愈率和生存率^[20]^，然而，在某些情况下，即使新辅助化疗降低了肿瘤分期，手术的复杂性也可能增加^[21]^。nICT组选择开胸方式比单纯手术组发生率高（P=0.002），但我们发现，nICT组中转开胸2例（16.67%），单纯手术组中转开胸2例（40.00%），而且，nICT组中选择开胸手术12例中8例患者手术发生在2021年及2022年初，这与当时临床医生对于NSCLC新辅助治疗后手术经验及手术方式选择上存在一定的偏倚，在临床实践中我们发现并没有增加手术难度。而且，我们发现nICT组与单纯手术组中的手术时间、术中出血量、带管时间、术后住院时间的差异均无统计学意义（P>0.05），但中位数值nICT组（162.50 min）相对于单纯手术组（180.00 min）要少，说明即使新辅助治疗后患者术中粘连较严重，但不影响手术操作，反而肿瘤降期对于局部NSCLC患者可以缩短手术中位时间且未增加术中出血量。故本研究认为nICT治疗不延迟手术时间和住院时间，并且可以提高R0切除率。

随着胸腔镜技术的发展，微创技术在NSCLC中的优势已被认可，如术中出血少、术后疼痛减轻、术后并发症率降低及死亡率低等^[22,23]^。本研究中，术后总并发症nICT组和单纯手术组分别为31.25%和22.64%，nICT组总并发症率略高于其他机构^[24,25]^，这可能与手术方式开胸率高有关。并发症中发生率最高的为肺部感染，其次为心律失常，这与李鹏飞^[26]^、廖小清^[27]^等结果相一致，且两组手术后并发症均无统计学差异（P>0.05）。因此，nICT治疗可切除局部晚期NSCLC不增加手术后并发症的发生率，具有可控的安全性和短期疗效的获益性。

nICT治疗提高了疗效，然而免疫治疗及化疗相关AEs也值得我们关注。本研究中所有患者均发生了不同程度的药物AEs，主要AEs为1和2级，发生率最高的AEs为脱发和乏力，3级AEs发生率为37.50%，发生率最高的为白细胞减少症。NADIM研究^[15]^中3级及以上治疗相关AEs发生率为30%；CheckMate816研究^[5]^中新辅助免疫化疗组AEs发生率为92.6%，3-4级AEs发生率为33.5%。本研究中，3级AEs发生率稍微高于其他研究，可能与临床过程中预防性用药不同及各个药物相关AEs不同有关，值得肯定的是，患者在nICT治疗期间安全性较好，未出现患者因AEs而导致治疗中止或死亡。

目前，癌症治疗的关注点逐渐扩展至患者的生活质量。nICT的目的不仅是为了延长生存期、提高R0切除率，患者的生活质量同样重要。本研究29例患者完成了nICT治疗前后生活质量的评估，其中，患者疲乏、恶心与呕吐、失眠、经济困难、口腔溃疡、周围神经痛、脱发、手臂及肩膀痛在nICT治疗后症状加重，而气促、咳嗽、咯血在nICT治疗后得到改善，这与蒋华等^[28]^对于肺癌化疗患者生活质量的研究结果大致相同。在一项探索性KEYNOTE-189^[29]^预先指定患者生活质量的评估研究中，在新辅助帕博丽珠单抗联合化疗第21周时，患者的疼痛、呼吸困难有明显好转，提高了部分患者的生活质量。生活质量评估反映了患者身体、心理和社会等方面的主观指标，本研究中大多数患者的主要症状得到缓解，获得了更好的生活质量。

本研究nICT组32例，直接手术53例，入组患者病例数少，可能会产生一定的偏倚。目前为止，患者总生存期（overall survival, OS）尚未得到，后续需要持续随访，进一步探索nICT与单纯手术的生存及预后价值。目前，“夹心饼”式围手术期免疫治疗模式^[30]^，不仅仅需要关注术前、术中的疗效及安全性，更应该注重患者术后的管理及预后。截止到目前，III期研究正在进行中^[31⇓-33]^，NEOTORCH研究^[31]^开创了全球首个“3+1+13”NSCLC围手术期治疗模式，特瑞普利单抗组接受手术的患者pCR达到24.8%，特瑞普利单抗组患者显著延长无病生存期（disease-free survival, DFS），存在OS获益趋势。RATIONALE-315研究^[34]^中采用“术前新辅助+术后辅助”围手术期治疗，pCR高达41%，虽然EFS、OS尚未达到，但都显示出获益趋势。因此，我们需要规范术后辅助方案及定期随访工作，加强患者术后辅助管理。

综上，nICT治疗局部晚期可切除NSCLC显示出良好的病理学缓解，未见明显增加术后并发症，且缓解了患者的临床症状，PD-1抑制剂可作为局部晚期可切除NSCLC患者nICT治疗的选择，但我们需要注意患者免疫化疗相关的不良反应，此外，本研究希望能够为III期临床试验奠定一定的基础，我们期待更多大样本、多中心、前瞻性的III期研究结果的公布。
